# Supplementary material for: The interactome of intact mitochondria by cross-linking mass spectrometry provides evidence for coexisting respiratory supercomplexes
Source: Mol Cell Proteomics. 2017 Dec 8;17(2):216–32. doi: 10.1074/mcp.RA117.000470 (PMC5795388; doi:10.1074/mcp.RA117.000470)
Supplement: Supplemental Data [file supp_RA117.000470_133922_0_supp_23978_fzffwf.zip › spectra_annotation/mito_spectra_annotation/342-1-3-1-1-1.pdf]

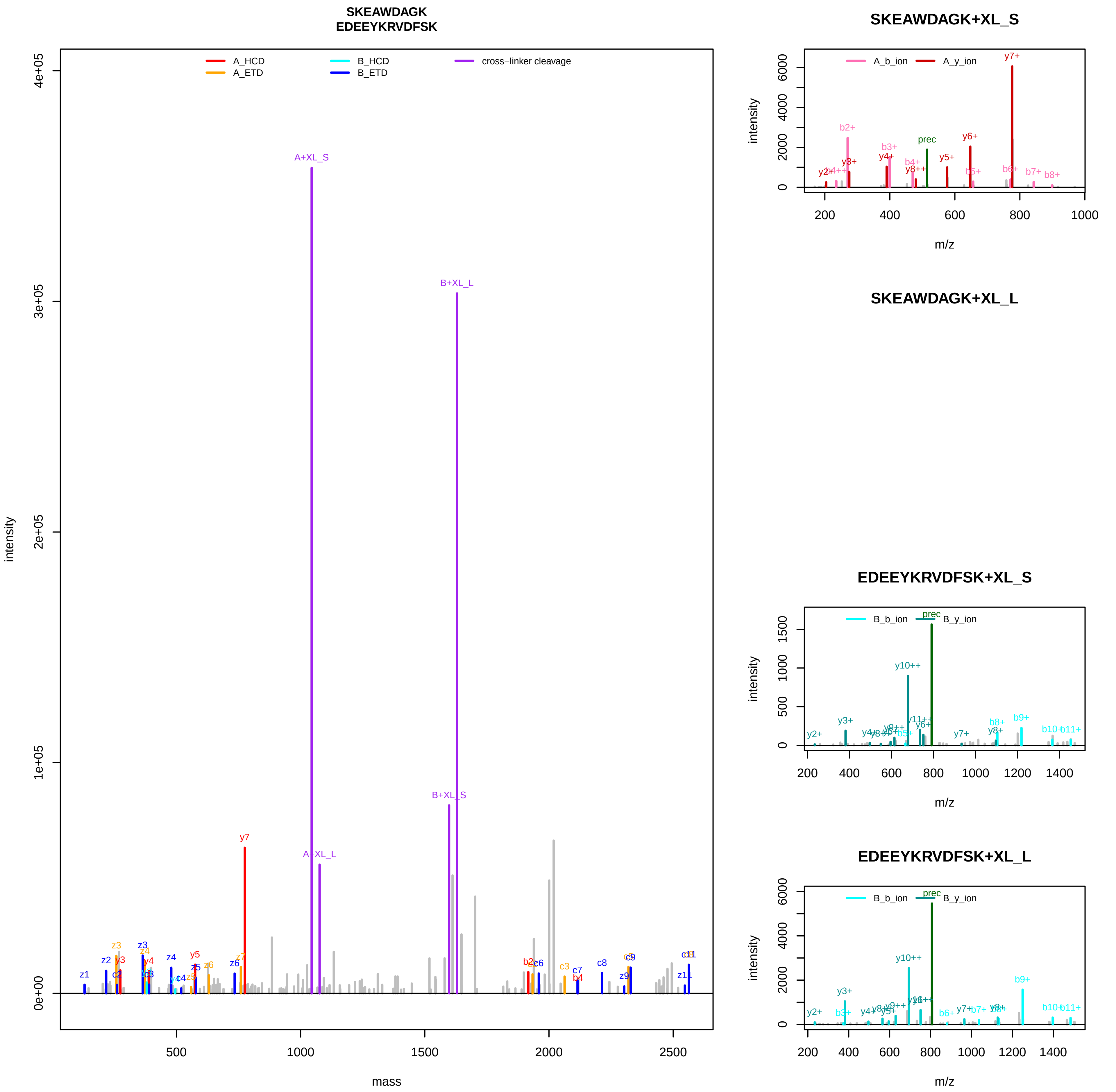
SKEAWDAGK
EDEEYKRVDFSK
A_HCD
A_ETD
B_HCD
B_ETD
cross−linker cleavage
A+XL_S
B+XL_L
B+XL_S
A+XL_L
y7
intensity
mass
0e+00
1e+05
2e+05
3e+05
4e+05
500
1000
1500
2000
2500
SKEAWDAGK+XL_S
A_b_ion
A_y_ion
y7+
b2+
prec
y6+
y5+
y4+
y3+
y2+
b3+
b4+
y8++
b5+
b6+
b7+
b8+
m/z
SKEAWDAGK+XL_L
EDEEYKRVDFSK+XL_S
B_b_ion
B_y_ion
prec
y10++
y3+
y2+
b8+
b9+
y8+
y7+
b10+
b11+
EDEEYKRVDFSK+XL_L
prec
y10++
y3+
y2+
b9+
b10+
b11+
m/z
